# Bioluminescent *Vibrio fischeri* Assays in the Assessment of Seasonal and Spatial Patterns in Toxicity of Contaminated River Sediments

**DOI:** 10.3389/fmicb.2016.01738

**Published:** 2016-11-07

**Authors:** Sergio Jarque, Petr Masner, Jana Klánová, Roman Prokeš, Ludek Bláha

**Affiliations:** Faculty of Science, Research Centre for Toxic Compounds in the Environment (RECETOX), Masaryk UniversityBrno, Czech Republic

**Keywords:** sediment, *Vibrio fischeri*, toxicity, monitoring, seasonality

## Abstract

Several bacteria-based assays, notably *Vibrio fischeri* luminescence assays, are often used as environmental monitoring tool for toxicity in sediments that may serve as both sinks and secondary source of contamination in aquatic ecosystems. In this study, we used 30-s kinetic bioassays based on *V. fischeri* to evaluate the toxicity associated to sediments from five localities with different contamination inputs (Morava River and its tributary Drevnice River in the south-eastern part of the Czech Republic). Toxicity assessed as half maximal inhibitory concentration (IC_50_) over the course of a year-long sampling was compared in bottom sediments and freshly trapped particulate material. Standard approach based on testing of aqueous elutriates was compared with toxicity of whole sediments (contact suspension toxicity). Bottom sediments showed lower toxicity compared to freshly trapped suspended materials in all cases. On the other hand, standardized elutriates induced generally weaker effects than suspended sediments likely due to losses during the extraction process. Toxicity generally increased during winter reaching maximum peaks in early spring months in all five sites. Total organic carbon (TOC) was found to be highly correlated with toxic effects. Toxicity from sites with direct industrial and agricultural water inputs also correlated with concentrations of metals, polycyclic aromatic hydrocarbons (PAHs), and polychlorinated biphenyls (PCBs). Single time point sampling followed by the extraction and testing of elutriates, do not truly reflect the spatial and temporal variability in natural sediments and may lead to underestimation of ecotoxic risks.

## Introduction

Natural compounds and anthropogenic pollutants such as organic compounds, pesticides, and heavy metals released into the environment accumulate in ecosystems, notably freshwater bodies, and pose risk to endogenous organisms. The undesired effects range from alteration of natural microbial communities (Sheik et al., 2012) to physiological disorders in higher organisms. The first may lead to changes in the trophic levels, while the second may result in species reproductive impairment or even lethality (Gust, 2006; Ward et al., 2013). Therefore, it is crucial to detect potential signs of toxicity in the earliest stages when damages in aquatic ecosystems are presumably less harmful and reversible. At present, toxicity in water bodies, including rivers, can be evaluated by analyzing the structure and abundance of biological communities, by measuring concentrations of chemicals that can be related to contaminant bioavailability and adverse effects and/or by direct assessing of toxicity using bioassays. The first approach is typically time-consuming and expensive, while the chemical measurements can be hard to extrapolate into biological systems and may lead to misinterpretation. As a consequence, there is a current need for the development of simple and inexpensive bioassays for the rapid assessment of environmental risks including aquatic matrices.

Sediments constitute an important part of aquatic ecosystems (Wetzel, 2001). If water bodies contaminate, sediments serve as trap for toxicants and potential secondary source of contamination, for example after resuspension of particles (Eggleton and Thomas, 2004) or after application on soils as fertilizer (Smith et al., 2008). Although sediments are considered relatively stable compared to the water phase, spatial, and temporal variability in sediments have been reported in many studies (Hilscherová et al., 2010; Bednarova et al., 2013; Macikova et al., 2014). Population densities and related anthropogenic activities, both agricultural and industrial, affect seasonal, and temporal patterns of contamination in fluvial sediments (Grosbois et al., 2006). Moreover, several abiotic factors such as sediment composition and geochemical processes can also influence final concentrations of pollutants and their toxicity (Bláha et al., 2010; Hilscherová et al., 2010; Perrichon et al., 2014).

At present, there are several bioassays used as monitoring tool to assess toxicity in sediments. They include representatives from all trophic levels—algae as producers, invertebrates or vertebrates as consumers and bacteria in the role of decomposers (Ahlf et al., 2002; Tuikka et al., 2011; Giusto et al., 2014; Hafner et al., 2015). Among them, bacterial assays have become particulary popular because of being simple, fast and inexpensive, having good correlations with other toxicity assay responses (Parvez et al., 2006) including higher organisms (Kahru et al., 1996; Kaiser, 1998). Accordingly, bacteria bioluminescence inhibition tests represent usually the first choice to test toxicity in sediments. These tests are traditionally performed by studying extracts or aqueous elutriates (more suitable for aquatic assays) or directly exposing bacteria to contaminated matrices, i.e., contact tests (Brouwer et al., 1990). The contact testing procedures, however, may be affected by confounding factors such as bacteria adsorption on particle surfaces, presence of ammonia, sulfur or sulfides, physical interference of bioluminescence with color or particles or pH variations (Volpi Ghirardini et al., 2009). As a suitable alternative for testing of sediments kinetic bioassay with *Vibrio fischeri* (also known as Flash assay) has been suggested, which uses each sample as its own reference, so color correction is possible with minimal manipulation (Lappalainen et al., 2001; Bláha et al., 2010).

In the present study, we assessed the toxicity of sediment samples from five locations with different chemical inputs in the south-eastern part of the Czech Republic using *V. fischeri* bioluminescence Flash tests. The approach based on testing of aqueous elutriates was compared with toxicity of whole sediments (contact suspension toxicity). The toxicity was assessed in bottom sediments (longer-term accumulation of toxicants) and freshly trapped particulate material (recent contamination events), respectively. Seasonal variability in toxicity was evaluated throughout a complete year. The toxicity results were correlated with concentrations of traditionally studied environmental contaminants like metals, PAHs or organochlorine compounds.

## Materials and methods

### Sediment sampling sites

**Figure 1:**
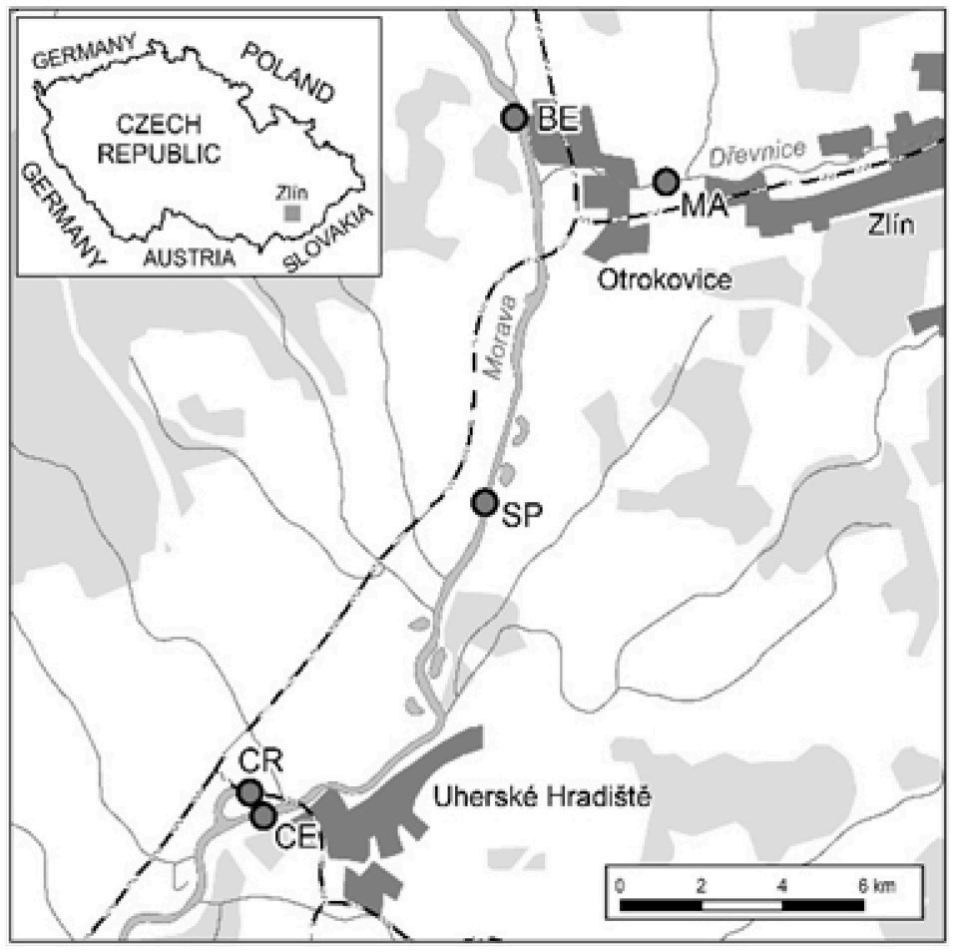
**Location of sampling sites within the studied area: MA, Malenovice (Drevnice River); BE, Belov (Morava River); SP, Spytihnev (Morava River); CE, Certak (Morava River); CR, Certak oxbow lake (Morava River)**.

In total 150 samples, 75 from bottom sediments, and 75 from recent sediments or sediment traps were collected from five different sampling sites (Malenovice, Belov, Spytihnev, Certak, and Certak oxbow lake) in the Morava River and its tributary Drevnice River, both located in the south-eastern part of the Czech Republic (Figure 1). Samples were periodically collected every 28 days from June 2007 to July 2008, with 15 samplings throughout the season. Near to Zlín city, Malenovice locality is situated on the Drevnice River in an area with strong presence of industries in rubber, footwear and mechanical engineering. Belov site is located in an agricultural area and reflects the situation in Morava River upstream from the confluence with Drevnice River. Spytihnev locality is situated downstream and integrates the contamination from both Morava and Drevnice rivers. Certak and Certak oxbow lake are both located on Morava River. Certak represents the normal watercourse, while Certak oxbow lake is part of a channel that remained abandoned since 1930s, when new channel regulations were implemented.

### Sample collection

Two types of samples, i.e., bottom sediments and fresh recent sedimented material were collected. Bottom sediments samples (top 0–10 cm layer) were collected using the plastic trowels. Recent sediments were taken using glass sediment traps placed close to the river bed but avoiding direct contact with bottom sediments. Unwanted debris such as large pieces of wood, leaves and stones were removed from all samples. The samples were then homogenized and subsequently freeze-dried (Gamma 1–16 LSC, Christ). Dry sediments were then sieved (2 mm mesh). Samples were clustered according to the four hydrological seasons, i.e., spring (March–May), summer (June–August), autumn (September–November), and winter (December–February), being October–March and April–September the colder and warmer seasons, respectively.

### Sediment characteristics

Sediment samples were characterized by measuring the content of total organic carbon (TOC) using high-temperature LiquiTOC II analyser (Elementar Analysensysteme). The grain size distribution was determined using a Retsch AS 200 sieving machine (Retsch, 0.036–4 mm fractions) and a Cilas 1064 laser diffraction granulometer (IVA Industrieberatung, 0.0004–0.5 mm fractions).

### Chemical analyses of organic contaminants

Freeze-dried sediments (10 g) were extracted with dichloromethane (DCM) using automated Büchi B-811 extractor (Büchi). The concentrated extracts were cleaned up on silica column (for PAHs) and H_2_SO_4_ modified silica column (organohalogens). Copper powder was used to remove sulfur. Samples were analyzed using GC-MS (Agilent 6890N GC/Agilent 5973N MS) supplied with a J&W Scientific fused silica column DB-5MS for 16 U.S. Environmental Protection Agency polycyclic aromatic hydrocarbons (PAHs), polychlorinated biphenyls (PCBs, congeners 28, 52, 101, 118, 138, 153, and 180), organochlorine pesticides (OCPs, namely α-, β-, γ-, and δ-isomers of hexachlorocyclohexane, HCH, p,p′-dichlorodiphenyltrichloroethane, p,p′-DDT, and metabolites p,p′-DDE and p,p′-DDD, and finally hexachlorobenzene, HCB). Concentrations of chemicals were quantified using Pesticide Mix 13 (Dr. Ehrenstorfer, GmbH) and PAH Mix 27 (Promochem) standard mixtures.

### Chemical analyses of metals

Concentrations of metals that are known to be toxic and represent environmental hazards in waters and sediments (V, Cr, Co, Ni, Cu, Zn, As, Mo, Cd, Sb, Pb, and Hg) were determined according to ISO 11466 method adapted to analytical instrumentation. Nitro-hydrochloric acid (2.3 ml HNO_3_ and 7.0 ml HCl) was used to leaching metals from sediments (1 g dry weight). Analyses were performed with using ICP-MS on Agilent 7500ce instrument (Agilent). Mercury in samples was determined with a thermo-oxidation method using AMA-254 analyser (Altec).

### Bacterial kinetic luminescence assay

Kinetic bioassays were performed in microplates as previously described (Bláha et al., 2010), with minor modifications. Given that the flash protocol is compatible with colored sediment matrices, toxicity in sediment samples was tested by direct contact between *V. fischeri* and the solid phase (Lappalainen et al., 1999, 2001). Sediment suspensions and aqueous elutriates were prepared according to European Norm (EN 12920:2006+A1:2008).

Sediment suspensions (100 mg dry wt sed./ml of distilled water, pH 7.0 ± 0.2) were pre-prepared by shaking (vortex, 2000 rpm) for 5 min and tested immediately or stored at 4°C for no more than 24 h before testing. Suspensions were then shaken once again (30 s) immediately before testing, and 0.15 ml were pipetted into micro-plate wells along with 0.01 ml of 32% w/v sodium chloride (NaCl) to reach the final concentration of 2% NaCl necessary for marine *V. fisheri*. Serial dilutions (1:1) for each sample were directly prepared in 2% w/v NaCl (pH 7.0 ± 0.2) in the microplate. Final concentrations tested (considering dilution with bacterial inoculum) were 75, 37.5, 18.75, 9.38, and 4.69 mg dry wt sed./ml. Elutriates were prepared in microtubes with sediment suspensions (100 mg dry wt sed./ml) shaken at 15 rpm for 24 h using a slow multi rotator. Suspensions were then centrifuged at 7000 rpm for 10 min and supernatants were diluted as previously described (serial dilutions 1:1 in 2% NaCl, 5 concentrations).

Microplates containing sample aliquots (0.08 ml of serial dilutions of sediment suspensions or elutriate supernatants), positive controls (K_2_Cr_2_O_7_, 380 mg/ml of 2% NaCl) and negative controls (2% w/v NaCl) were temperate at 15°C and toxicity tested as described below.

Freeze-dried luminescent bacteria *V. fischeri* NRRL B-11177 (Institute of Microbiology, Czech Academy of Sciences) were reconstituted according to ISO 11348-3 (1998) in ice-cold 2% w/v NaCl and kept on ice. Prior to testing, aliquots of bacterial suspension were diluted in 2% NaCl and tempered at 15°C in a water bath for 30 min. 0.02 ml of bacteria suspension were injected into each well of the microplates with studied samples, and the luminescence was immediately monitored for 2 s (initial peak value). The luminescence was measured using a microplate Luminoscan Ascent luminometer (Thermo) equipped with computer-controlled injectors. The microplate was then shaken inside the luminometer incubated at 15°C and the final toxicity signal recorded after 30 s (S30-value). The inhibition of luminescence (percentage of control) was calculated as follows:

$$\begin{array}{l} INH\%\;=\;100\;-\;\frac{\text{S}30}{CF\;\times \;Peak}\;\times \;100, \end{array}$$

where *CF* is a correction factor (the *S*30/peak ratio in negative controls) reflecting natural attenuation of bacterial luminescence during 30 s exposures.

Concentrations of sediment suspensions that caused 50% inhibition of luminescence (IC_50_ in mg dry weight/ml) were derived from the four-parametric logistic curve calculated in Graph-Pad™ Prism (GraphPad Software). Because sediment elutriates rarely caused 50% decreases in luminescence, the IC_50_-value was replaced in those cases by the INH_75_-value, which was defined as a decrease in the light emission observable after 30 s in a non-diluted aqueous extract corresponding to 75 mg dw sed./ml.

### Data analyses

Standard summary statistics were used to describe distributions of the primary data. Non-parametric analyses were applied, because assumptions of parametric analyses (normality of data distribution) were not found in all variants. Correlations between sediment parameters and toxicity were tested using Spearman's rank correlation, differences were tested by Wilcoxon paired test. For all of the tests, *p* < 0.05 were considered statistically significant. Calculations were performed in Statistica (StatSoft, Tulsa, OK, USA).

To better visualize the results, the individual data points within each analyzed parameters (for chemical analyses results as well as toxicity) were categoried into four groups (I–IV). This approach allowed for better relative comparisons of different analyzed parameters, where the actual numerical values ranged across orders of magnitude. Group I represented the smallest levels of contamination (or the lowest toxicity, respectively), and the group IV contained the highest values of contamination or toxicity, respectively. The groups I–IV were formed as follows: the minimum (MIN0) and maximum (MAX0) of all detected values of a certain parameter were cut off to eliminate extremes. The values falling between the second minimal (*MIN*) and the second maximal (*MAX*) values were then divided into four groups (I–IV) based on the statistical distributions. For each parameter (i.e., toxicity value, organic carbon-TOC, or concentrations of individual contaminants), the upper limits (L) for each of the 4 groups were calculated:

$$\begin{array}{l} L\;=\;MIN\;+\;a\cdot \frac{\left(MAX\;-\;MIN\right)}{4}, \end{array}$$

where *a* = 1 or 2 or 3 or 4 for Groups I–IV, respectively. Examples of criteria (limits, *L*) for groups I, II, III, and IV for selected parameters (TOC, sum of PAHs, total Cd concentrations) are shown in Supplementary Table S1.

## Results

Summary of the toxicity testing is shown in Table 1. Detailed results for all bottom and recent sediment samples are presented in Supplementary Table S2 (toxicity of suspended materials) and Supplementary Table S3 (the inhibitory effects observed in aquatic elutriates).

**Table 1:** **Annual and seasonal toxicity (IC_50_) levels obtained with the 30-s kinetic *Vibrio fischeri* assay in bottom and recent sediments tested in suspensions**.

	**Locality^b^**	**N^c^**	**Toxicity (IC_50_, mg/ml)^a^**				
			**Spring**	**Summer**	**Autumn**	**Winter**	**All seasons**
**Bottom sediment**		**73 (15 + 24 + 15 + 19)**	**53.6 (12.4 – > 75.0)**	**>75.0 (30.6 – > 75.0)**	**63.2 (36.5 – > 75.0)**	**55.8 (13.5 – > 75.0)**	**63.8 (12.4 – > 75.0)**
	MA	15	39.4	71.0	60.6	23.6	43.8
		(3 + 5 + 3 + 4)	(12.4 – 74.1)	(30.6 – > 75.0)	(36.5 – 65.0)	(13.5 – 43.8)	(12.4 – > 75.0)
	BE	14	27.9	72.5	41.9	34.4	46.1
		(3 + 4 + 3 + 4)	(20.1 – 34.9)	(63.3 – > 75.0)	(37.6 – 54.3)	(19.2 – 58.3)	(19.2 – > 75.0)
	SP	14	53.6	60.5	62.1	73.9	60.9
		(3 + 5 + 3 + 3)	(38.5 – 58.3)	(49.1 – 63.8)	(61.2 – 63.2)	(46.4 – > 75.0)	(38.5 – > 75.0)
	CE	15	>75.0	>75.0	>75.0	>75.0	>75.0
		(3 + 5 + 3 + 4)	(>75.0 – > 75.0)	(>75.0 – > 75.0)	(70.3 – > 75.0)	(>75.0 – > 75.0)	(70.3 – > 75.0)
	CR	15	54.4	>75.0	>75.0	67.9	>75.0
		(3 + 5 + 3 + 4)	(25.4 – > 75.0)	(>75.0 – > 75.0)	(>75.0 – > 75.0)	(48.0 – > 75.0)	(25.4 – > 75.0)
**Recent (trap) sediment**		**49 (10 + 16 + 10 + 13)**	**26.0 (4.6 – 53.7)**	**43.5 (10.4 – 63.7)**	**29.6 (18.0 – 75.8)**	**21.2 (4.9 – > 75.0)**	**28.4 (4.6 – > 75.0)**
	MA	12	7.8	38.2	22.3	8.1	15.7
		(2 + 4 + 3 + 3)	(4.6 – 11.0)	(10.4 – 56.0)	(20.3 – 37.7)	(4.9 – 9.4)	(4.6 – 56.0)
	BE	9	30.8^d^	42.3	39.1	27.5	30.8
		(1 + 3 + 2 + 3)		(41.9 – 42.5)	(23.1 – 55.2)	(21.2 – 27.6)	(21.2 – 55.2)
	SP	11	27.6	50.0	36.3	20.5^d^	33.5
		(3 + 4 + 3 + 1)	(24.5 – 29.8)	(33.5 – 63.0)	(30.5 – 75.8)		(20.5 – 75.8)
	CE	7	53.7^d^	54.5	28.8^d^	45.7	45.7
		(1 + 2 + 1 + 3)		(45.4 – 63.7)		(21.7 – > 75.0)	(21.7 – > 75.0)
	CR	10	19.4	33.1	18.0^d^	14.7	18.7
		(3 + 3 + 1 + 3)	(17.3 – 28.4)	(16.9 – 48.8)		(14.2 – 22.9)	(14.2 – 48.8)

a Values indicate concentrations causing 50% inhibition of bioluminescence after 30-s exposures (mg sediment dry weight/ml). Expressed as median (min—max).

b MA, Malenovice; BE, Belov; SP, Spytihnev; CE, Certak; CR, Certak oxbow lake.

c Number of samples—total number and numbers from different seasons (Spring + Summer + Autumn + Winter).

d The only detected value.

### Toxicity of sediment suspensions vs. aqueous elutriates

In general, aquatic elutriates were significantly less toxic compared to the suspensions from the same sampling site. In elutriates the 50% inhibitory effects were observed only rarely, and the toxicity could be described only as inhibitions of luminescence (%) at the highest tested concentration of sediment elutriate (corresponding to 75 mg dw/mL, Supplementary Table S3). Sediment suspensions showed in general more pronounced effects allowing for calculation of IC_50_ (Supplementary Table S2 and Table 1). However, the toxic responses of *V. fisheri* to elutriates and suspensions were significantly correlated (Spearman rank coefficient for all samples *N* = 121, *Rs* = 0.849, *p* < 0.0001).

### Toxicities of bottom sediments vs. fresh sediment from traps

Detailed investigation of the toxicity results of suspended materials revealed that recent sediments from traps (IC_50_ median values ranging from 7.8 to 53.7 mg dry weight/ml) were generally more toxic than bottom sediments (IC50 23.6 to >75 mg dry weight/ml; Table 1). The difference was confirmed by Wilcoxon Matched Pairs Test (*N* = 47, *Z* = 5.46, *p* < 0.0001).

### Seasonal variability in toxicity responses

**Figure 2:**
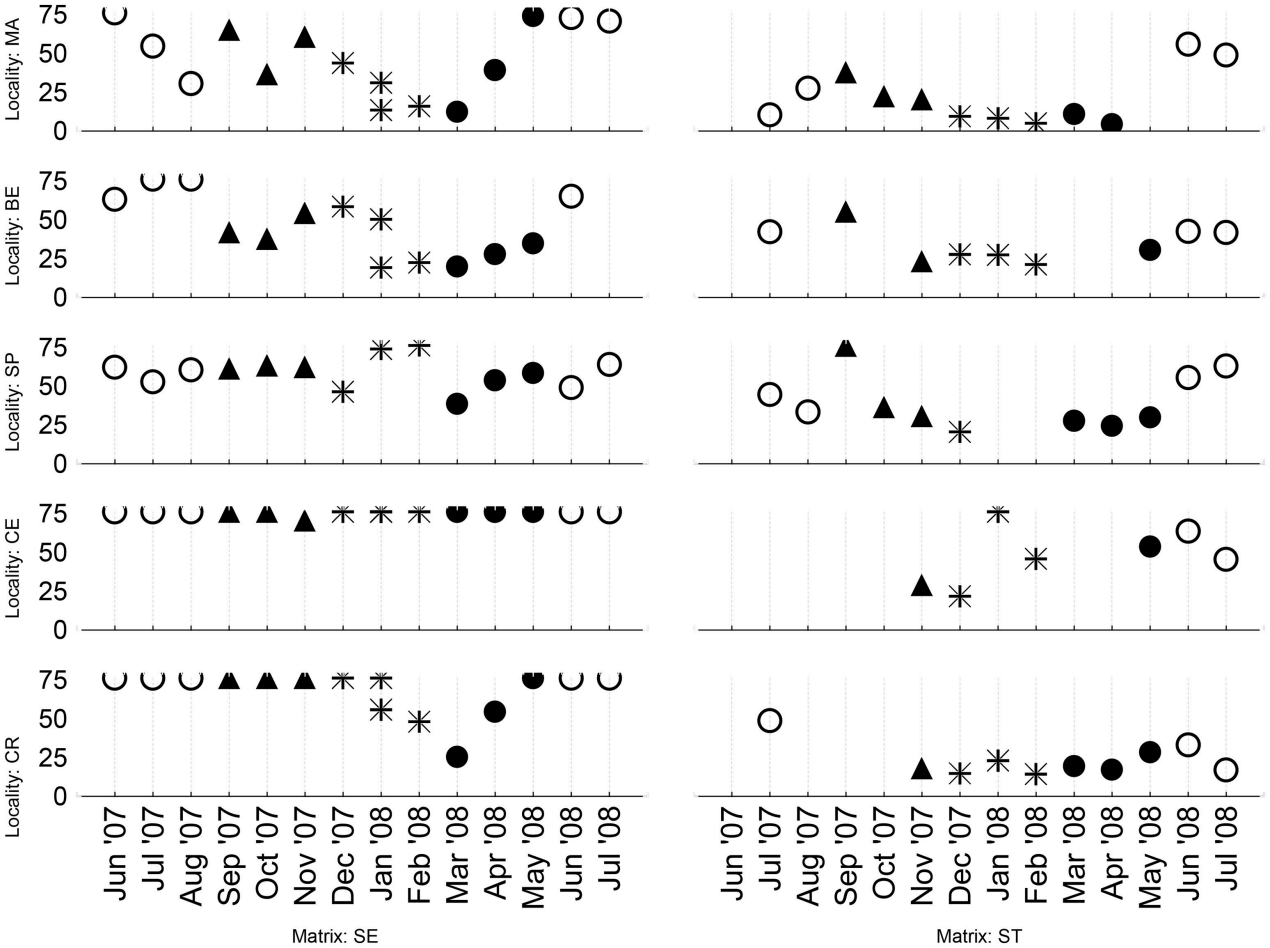
**Seasonal toxicity in samples of bottom sediment (SE—left graphs) and samples from sediment traps (ST) at given sampling sites/localities (MA, Malenovice; BE, Belov; SP, Spytihnev; CE, Certak; CR, Certak oxbow lake)**. The toxicity (expressed as 50% inhibition concentration, IC50, in mg dry wt sediment/ml) is pictured vertically, whereas the horizontal axis shows the date of sampling (N.B. two samplings were carried out in January—on the 2nd and 30th). Used symbols correspond to clustering of samples into four seasons (● Spring, ◯ Summer, ▴ Autumn, 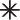 Winter).

Toxicity of both bottom and freshly trapped sediments showed a clear seasonal pattern for all sampling sites (Table 1, Supplementary Table S2, Figure 2). The toxicity increased during the winter period and reached the lowest IC50-values at the end of February or beginning of March. Systematically lowest toxicity was detected during summer months.

### Spatial variability in toxicity

**Figure 3:**
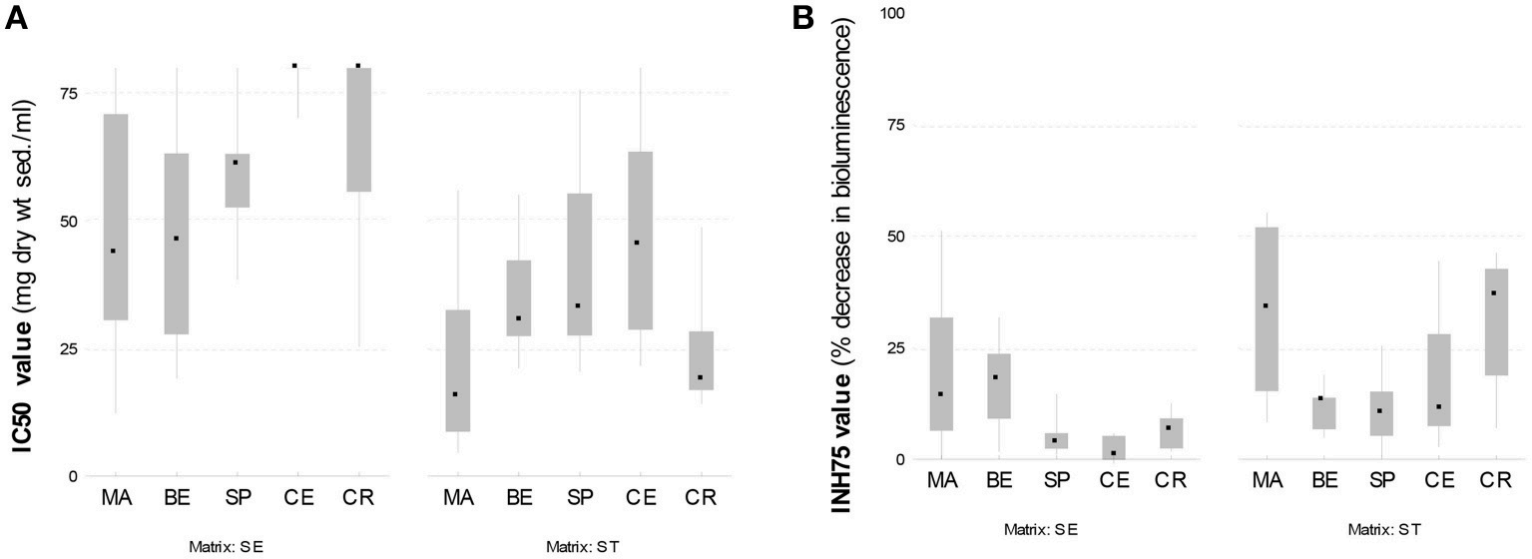
**Spatial distribution of toxicity—IC50-values from contact testing (A) and INH75-values from elutriate testing (B)—in bottom sediment (SE) and recent sediment (ST)**. MA, Malenovice; BE, Belov; SP, Spytihnev; CE, Certak; CR, Certak oxbow lake.

**Figure 4:**
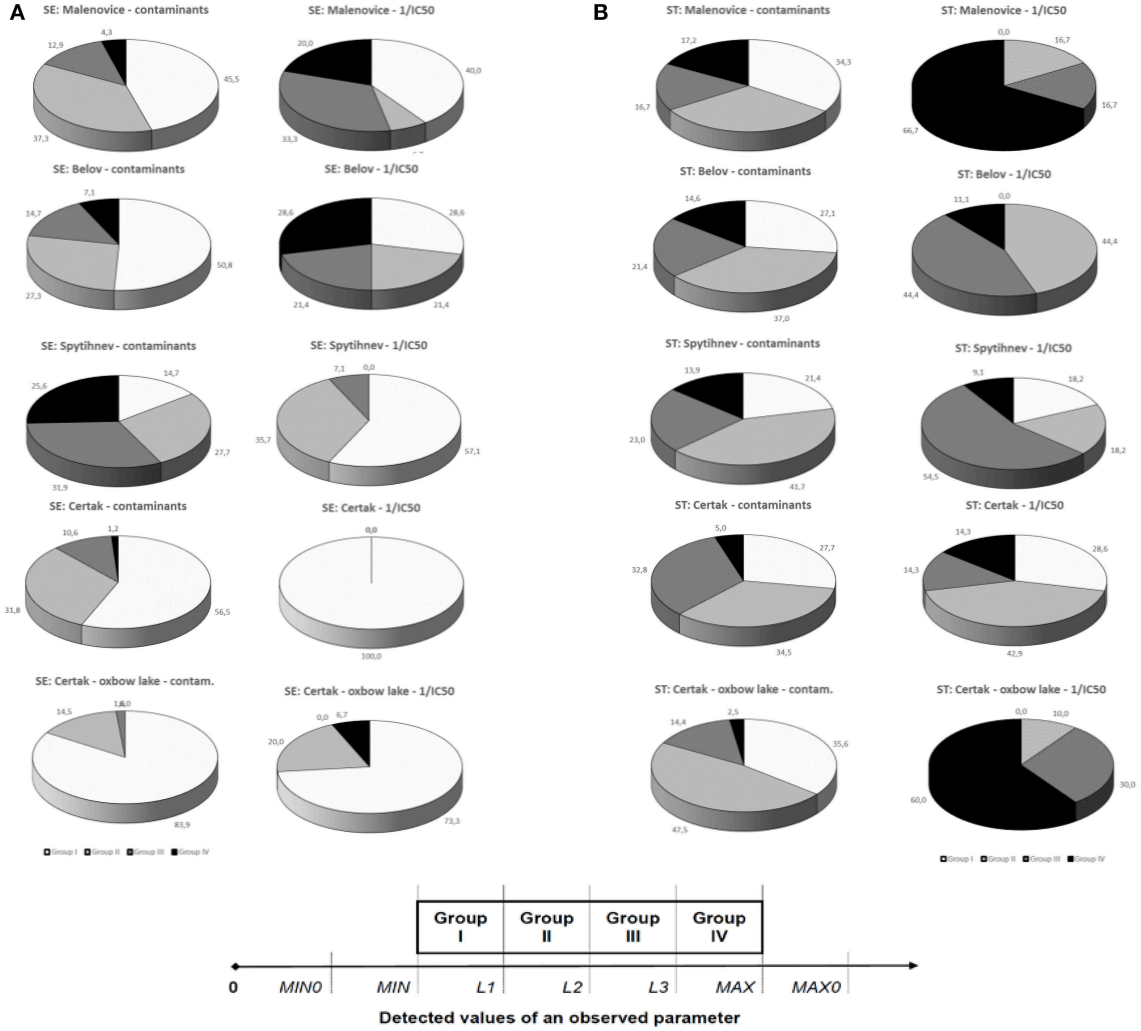
**Comparison of relative contamination (a collation for 17 groups of chemical classes) of bottom (SE, part A) and recent (ST, part B) sediment and relative toxicity detected with 30-s kinetic *Vibrio fischeri* assay**. Group I (white sector) represents the lowest degree of contamination/toxicity, Group IV (black sector) the highest one. Numbers express a percentage of all values in given group. Criteria for grouping of detected values for each contaminant are described in Section Materials and Methods in more detail. Scheme of relative contamination clustering: MIN0, the minimal value from all detected values; MIN, the second minimal value from all detected values; L1, the upper limit for the 1st group; L2, the upper limit for the 2nd group; L3, the upper limit for the 3rd group; MAX, the second maximal value from all detected values; MAX0, the maximal value from all detected values.

With respect to bottom sediments, the lowest toxicity was systematically found at Certak and Certak oxbow lake, the two sites further downstream away from the city of Zlín (the highest IC50-values in Table 2 and Figure 3A). The pattern is also clear from Figure 4A—bottom sediments), where distributions of 1/IC50 toxicity values are shown as pie charts. On the other hand relatively higher toxicities of bottom sediments were found at Malenovice and Belov sites. For fresh sediments from traps (Figures 3B, 4B), toxicity results did not show as apparent spatial differences between sites as for bottom sediments. The site with the highest toxicity was again Malenovice and interestingly, the second highest toxicities were found at Certak oxbox-lake.

**Table 2:** **Correlation between the toxicity (expressed as 1/C50 for solid phase test and INH75 for elutriate test—both tested with the 30-s kinetic *Vibrio fischeri* assay) and selected sediment parameters and concentrations of contaminants in bottom sediment (SE)**.

	**1/IC50 (solid phase)**						**INH75 (elutriates)**					
**Locality^a^ (n)^b^**	**MA (15)**	**BE (14)**	**SP (14)**	**CE^d^ (15)**	**CR (15)**	**All (73)**	**MA (15)**	**BE (14)**	**SP (14)**	**CE (15)**	**CR (15)**	**All (73)**
TOC^c^	0.525	0.946	NS	NS	0.568	0.668	0.782	0.837	0.578	NS	NS	0.553
PAHs	NS	0.889	NS	NS	NS	0.396	0.579	0.855	NS	NS	NS	NS
PCBs	0.577	0.661	NS	NS	NS	0.391	0.683	0.810	NS	NS	NS	NS
HCHs	NS	NS	NS	NS	NS	0.346	NS	0.534	NS	NS	NS	0.289
DDTs	NS	0.621	NS	NS	NS	0.400	NS	0.774	NS	NS	NS	NS
HCB	NS	NS	NS	NS	NS	0.375	0.680	0.635	NS	NS	NS	0.338
V	NS	NS	NS	NS	0.655	0.565	0.632	NS	NS	NS	NS	0.364
Cr	NS	NS	NS	NS	0.623	NS	0.634	0.574	NS	NS	NS	NS
Co	0.621	0.581	NS	NS	0.674	0.537	0.821	0.714	NS	NS	NS	0.341
Ni	0.600	0.537	NS	NS	0.660	0.529	0.804	0.675	NS	NS	NS	0.307
Cu	0.693	0.670	NS	NS	NS	0.522	0.943	0.780	NS	NS	NS	0.301
Zn	0.689	0.678	NS	NS	NS	0.489	0.929	0.776	NS	NS	NS	0.259
As	NS	0.695	NS	NS	0.596	NS	0.525	0.802	NS	NS	NS	NS
Mo	0.643	0.876	NS	NS	0.706	0.680	0.825	0.943	NS	NS	NS	0.393
Cd	NS	0.942	NS	NS	NS	0.317	0.743	0.881	NS	NS	NS	NS
Sb	NS	0.662	NS	NS	NS	0.308	0.675	0.653	NS	NS	NS	NS
Pb	0.582	0.880	NS	NS	NS	0.496	0.700	0.912	NS	NS	NS	NS
Hg	0.568	0.942	NS	NS	NS	0.302	0.847	0.877	NS	NS	NS	NS

a MA, Malenovice; BE, Belov; SP, Spytihnev; CE, Certak; CR, Certak oxbow lake; All, all sampling sites.

b Number of paired values for correlation.

c TOC, total organic carbon (%); PAHs, sum of polycyclic aromatic hydrocarbons (ng/g); PCBs, sum of polychlorinated biphenyls (ng/g); HCHs, hexachlorocyclohexane (all congeners, ng/g); DDTs, DDT and its metabolites (ng/g); HCB, hexachlorobenzene (ng/g); 1/IC50, inverse value of 50% inhibition concentration derived from the kinetic V. fischeri assay (mg dry wt/ml); INH75, decrease in light emission caused by 75 mg dry wt sediment in test with elutriates (%).

d Correlation is not possible here, because all toxicity values were >75 mg dry wt/ml.

R-values of Spearman rank correlation that were statistically significant (P < 0.05) are shown. (NS, no significant correlation).

### Contamination results

The complete results of analysis of total 17 different contaminant groups—five organic (sums of PAHs, PCBs, HCHs, DDTs, and HCB) and 12 toxic metals in studied bottom and fresh sediments are shown in Supplementary Table S4 (EXCEL). To better visualize and compare the chemical loads, the values were transferred to relative groups I–IV as described in Materials and Methods Section, and the aggregate results per individual sites are shown in Figure 4A (bottom sediments) and Figure 4B (sediment traps). Bottom sediments were most polluted at Spytihnev site (almost 60% of the values were in the highest contamination Groups III or IV—cf Figure 4A—Spytihnev pie charts for contamination). Interestingly, the samples from this site did not elicit major toxic effects (cf Figure 4A—piecharts for 1/IC50-values). On the contrary, the lowest levels of bottom sediment contamination were detected in Certak oxbow lake, and the findings corresponded to the pattern of toxicities (cf Figure 4A). With regard to freshly trapped sediments (Figure 4B) no major differences were apparent among the sites in contaminant levels or toxicity (as apparent from the distribution patterns shown in pie-charts).

### Correlations between toxicity and contamination

Correlation analyses between the toxicity values (1/IC_50_ for solid phase test and INH_75_ for elutriates) and 17 contaminant parameters and total organic carbon (TOC) are shown in Table 2 (bottom sediment samples) and Table 3 (sediment traps).

**Table 3:** **Correlation between the toxicity (expressed as 1/C50 for solid phase test and INH75 for elutriate test—both tested with the 30-s kinetic *Vibrio fischeri* assay) and selected sediment parameters and concentrations of contaminants in *recent* sediment (ST)**.

	**1/IC50 (solid phase)**						**INH75 (elutriates)**					
**Locality^a^ (n)^b^**	**MA (12)**	**BE (9)**	**SP (11)**	**CE (7)**	**CR (10)**	**All (49)**	**MA (12)**	**BE (9)**	**SP (11)**	**CE (7)**	**CR (10)**	**All (49)**
TOC^c^	0.858	NS	0.764	NS	NS	0.643	0.869	0.717	0.800	0.793	NS	0.614
PAHs	0.776	NS	NS	NS	NS	NS	0.727	NS	0.618	NS	NS	0.294
PCBs	0.783	0.933	NS	0.899	NS	0.607	0.825	0.883	NS	NS	NS	0.664
HCHs	NS	NS	NS	0.943	NS	NS	NS	NS	NS	0.928	NS	NS
DDTs	NS	NS	NS	0.829	NS	NS	NS	NS	−0.690	NS	NS	NS
HCB	0.603	0.750	NS	0.886	NS	NS	0.667	NS	NS	0.841	NS	NS
V	NS	NS	NS	NS	NS	NS	NS	NS	NS	NS	NS	−0.407
Cr	NS	NS	NS	NS	−0.636	−0.389	NS	NS	−0.664	NS	−0.636	−0.514
Co	NS	NS	NS	0.893	NS	NS	0.620	NS	NS	0.829	NS	NS
Ni	0.622	NS	NS	0.893	NS	NS	0.825	NS	NS	0.829	NS	NS
Cu	0.790	NS	NS	0.821	NS	0.531	0.902	0.683	NS	NS	NS	0.405
Zn	0.671	0.733	0.636	0.929	NS	0.604	0.699	0.850	NS	0.901	NS	0.487
As	NS	NS	NS	NS	NS	−0.373	NS	NS	NS	NS	NS	−0.535
Mo	0.895	NS	NS	NS	NS	0.303	0.874	0.667	0.791	NS	NS	NS
Cd	0.692	0.800	NS	NS	NS	NS	0.671	0.933	NS	0.883	NS	NS
Sb	0.685	NS	NS	NS	NS	NS	0.629	NS	NS	NS	NS	NS
Pb	0.664	0.817	NS	NS	NS	NS	0.671	0.683	NS	NS	NS	NS
Hg	0.629	NS	NS	NS	NS	0.345	0.643	NS	NS	NS	NS	NS

a MA, Malenovice; BE, Belov; SP, Spytihnev; CE, Certak; CR, Certak oxbow lake; All, all sampling sites.

b Number of paired values for correlation.

c TOC, total organic carbon (%); PAHs, sum of polycyclic aromatic hydrocarbons (ng/g); PCBs, sum of polychlorinated biphenyls (ng/g); HCHs, hexachlorocyclohexane (all congeners, ng/g); DDTs, DDT and its metabolites (ng/g); HCB, hexachlorobenzene (ng/g); 1/IC50, inverse value of 50% inhibition concentration derived from the kinetic V. fischeri assay (mg dry wt/ml); INH75, decrease in light emission caused by 75 mg dry wt sediment in test with elutriates (%).

R-values of Spearman rank correlation (NS, no significant correlation).

In bottom sediments (Table 2), 16 out of 18 variables correlated with the IC_50_-values from tests with solid phase suspensions when considering data from all sites (*N* = 75). The toxicity of elutriates (INH75-values) correlated with only eight parameters. In the sediments from Malenovice and Belov (where the toxicity values were variable and widely distributed—cf Figure 2A) the highest number of significant correlations was observed for both 1/IC50 and INH75 (especially between the toxicity and metal concentrations, Table 2). Only rare correlations were detected at the other sites. For recent sediments from traps (Table 3), generally lower number of significant correlations between toxicity and matrix composition was recorded. Correlations in samples from Malenovice site highlighted links to concentrations of PAHs, PCBs, HCB, and some metals.

**Figure 5:**
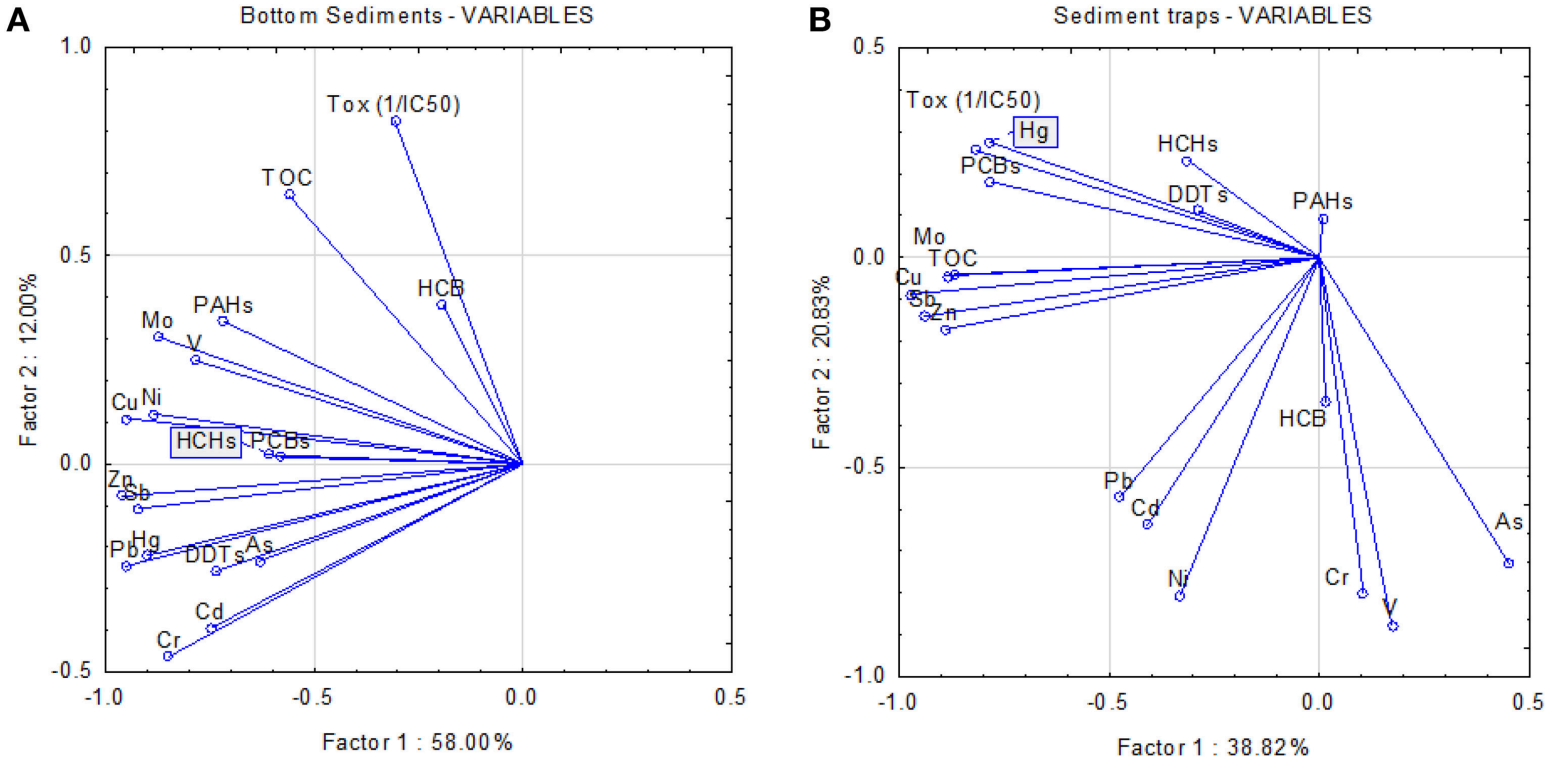
**Results of the principal component analysis (PCA) represented as score plots for factor 1 and factor 2 for bottom sediments (panel A) and fresh material from sediment traps (B)**. The 18 parameters [toxicity (Tox (1/IC50)) and chemical compounds] are shown as variables.

Further, principal component analysis (PCA) defined two principal factors that explained the 70 and 59.6% of the total observed variability in bottom sediments and trap samples, respectively (Figure 5). Toxicity in bottom sediments appeared to be intimately related to TOC, although close associations were also found for HCB and PAHs. Heavy metals seemed to not contribute to the toxic activity. The relationship between toxicity and TOC was also apparent in sediment traps, but this association was slightly less pronounced compared to bottom sediments. Persistent pollutants such as PCBs, DDT, and HCH seemed to also contribute to toxicity. Similar to bottom sediments, toxicity seemed not to be related to the presence of metals.

## Discussion

*V. fischeri* kinetic tests were successfully applied to assess toxicity in sediment suspensions and aqueous elutriates from five different sites in the Morava river basin, Czech Republic, which is a representative model ecosystem for mid-size industrial cities in central Europe. Although toxicity values for both approaches (suspension test vs. elutriate) showed a good correlation, toxicity in elutriates was significantly lower. This is in line with previous studies that described reduced toxicities of the extracts (compared to whole sediments) due to the absence of particle-bounded toxicants and poor extraction of nonpolar compounds such as PCBs and PAHs (Ho and Quinn, 1993; Harkey et al., 1994; Guzzella, 1998). For this reason, testing of bacterial toxicity of elutriates can be recommended for water-soluble compounds rather than to reflect the total toxicity of bulk sediment (Ankley et al., 1991).

Compared to bottom sediments, recently collected (trap) sediments showed nearly always (91.5% of all samples) higher toxicity at all sampling sites, but no significant differences were observed among locations. On the contrary, bottom sediments generally showed higher toxicity at upstream sites located in a closer proximity to direct contamination sources. This is in agreement with recent studies of marine sediments, where different toxicity profiles were found between coastal/estuarine sediments and offshore sediments (Vethaak et al., in press). Interestingly, the most contaminated sites not always showed the highest toxicity in *Vibrio* assays. For instance, sediments from Spytihnev were highly contaminated compared to the rest of locations, while toxicity ranked among the lowest. This no linear correlation between chemical burden and toxicity has been described in several studies and points out to additional factors as toxicity drivers. In this sense, differences in bacterial toxicity may be related to the particular composition of the sediment and the different chemical association to sediment particles (Elzerman and Coates, 1987; Baker et al., 1991). It is well-known that hydrophobic pollutants present different sorption behavior on silt and lime fractions, which determines their physical movement, bioavailability, persistence, and degradation, and that the differences in sorption depend largely on the amount of total organic carbon within each fraction (Karickhoff et al., 1979). For instance, strong positive relationships between TOC and PAHs (Guo et al., 2009), metals (Chakraborty et al., 2014), and PCBs content (Ayris and Harrad, 1999; Backe et al., 2004) have been previously reported in sediments. Similarly, our results reflect a good correlation between TOC and the observed toxicity in the most toxic sites, pointing out TOC as a major candidate as toxicity driver. However, a linear relationship between TOC and chemical burden, e.g., organochlorine pesticides, is not always apparent (Parween et al., 2014), and therefore other sediment characteristics such as exchangeable H+ or sulfur content could play an important role in the final sediment effect toxicity detected in our sediment samples (Hilscherova et al., 2007; Hilscherová et al., 2010). To our knowledge there are no evidences or studies indicating direct cytotoxic effects of TOC. However, major components of TOC such as humic acids have previously been shown to interfere with more subtle biological endpoints such as intracellular receptors in eukaryotic cells (Bittner et al., 2006; Janošek et al., 2007). Therefore, the relevance of biological effects of TOC and their eventual toxic outcomes should further be investigated.

POPs content in Morava river sediments was recently characterized (Prokeš et al., 2014; Supplementary Table S4). Similar to other rivers in Central Europe, PAHs are the dominant group of contaminants in the area (Müller et al., 2002). In contrast, concentrations of heavy metals are rather low to moderate with only few local exceptions (Bednarova et al., 2013, this study). Organochlorine pesticides and PCBs seem to present a more diffusive contamination probably reflecting a long-range transport contamination rather than local emissions. In fully agreement, results obtained from PCA disclosed important contributions in the overall toxicity for HCB and PAHs in bottom sediments, and PCBs, DDT, and HCH in sediment traps. On the contrary, heavy metals appeared to have a negligible role. Taking all together, PAHs are the compounds more likely to induce toxicity in our studied samples. Consistent with our hypothesis, lower chlorinated PCBs and lighter PAHs have been described to rapidly removed from surface waters and settle through the water column to the sediment water interface, easily mixing back into the water column. Conversely, PAHs with high molecular weights settle more slowly, getting buried in the sediments and not mixing with waters (Baker et al., 1991).

Toxicity in sediments followed seasonal patterns in all cases, increasing during winter, reaching its toxic peak in spring and decreasing in summer. Various biotic and abiotic factors such as human activities, temperature, rainfall, or drought may contribute to modulate, either positively or negatively, this temporal toxic effect (Müller et al., 2002; Stachel et al., 2005; Rosado et al., 2016). However, recent studies in POPs content and annual dynamics in this area pointed out the water flow as one of the major potential causes to explain the observed seasonal variation (Prokeš et al., 2014). Chemical concentrations in sediments would be modulated by episodic high flow events provoking the erosion of contaminant-containing particles. This would result in (i) resuspension of chemicals and thus increase of their bioavailability, particularly in recent sediments, and (ii) movement of certain chemicals from sources to downstream sites. Both processes would increase toxicity associated to those chemicals, notably PAHs (Stachel et al., 2005; Bláha et al., 2010; Prokeš et al., 2014). In addition, TOC in sediments typically increases during springtime. Sediments with higher concentrations of TOC have a larger sorption capacity for hydrophobic chemicals, which affects bioavailability of these pollutants. *V. fischeri* kinetic assays were able to reflect these temporal changes, highlighting the importance of periodical biological (effect-based) monitoring to avoid misinterpretation associated to specific time points.

## Conclusions

The *V. fischeri* kinetic assay was proved to be a suitable tool for the assessment of toxicity in solid and colorful samples such as fluvial sediments. The study confirmed the good correlation between the *V. fischeri* kinetic assay performed directly in sediments (contact test) and the toxicity values obtained in elutriates. Our study demonstrated higher toxicity in samples from sediment traps in comparison to samples from bottom sediment collected at the same locality in the same time. In full agreement with previous hydrodynamics-based studies in the same area (Prokeš et al., 2014), toxicity showed clear seasonal patterns, typically increasing during winter and decreasing in summer, which highlights the importance of monitoring throughout the year rather than specific time points. Given the high concentrations of PAHs, the most dominant group of pollutants in the area, and the good correlation with both toxicity and TOC, PAHs seem to be the primary cause of toxicity. However, moderate contribution of other compounds such as organochlorine pesticides or metals (or other not analyzed contaminants) cannot be ruled out. Although the lack of standardized safety guidelines may limit the applicability of *V. fischeri* kinetic assays in sediments, some guidance values for acute toxicity have been recently suggested (Bláha et al., 2010) promoting thus further use of effect-based monitoring of environmental quality.

## Author contributions

LB coordinated the research; LB and JK designed the study; PM, JK, RP performed research and analyzed data; SJ integrated and interpreted the results and wrote the paper, all coauthors contributed to paper finalization.

### Conflict of interest statement

The authors declare that the research was conducted in the absence of any commercial or financial relationships that could be construed as a potential conflict of interest.
