# Supplementary material for: Bioluminescent Vibrio fischeri Assays in the Assessment of Seasonal and Spatial Patterns in Toxicity of Contaminated River Sediments
Source: Front Microbiol. 2016 Nov 7;7:1738. doi: 10.3389/fmicb.2016.01738 (PMC5097916; doi:10.3389/fmicb.2016.01738)
Supplement: Supplementary file 1 [file Table1.DOCX]

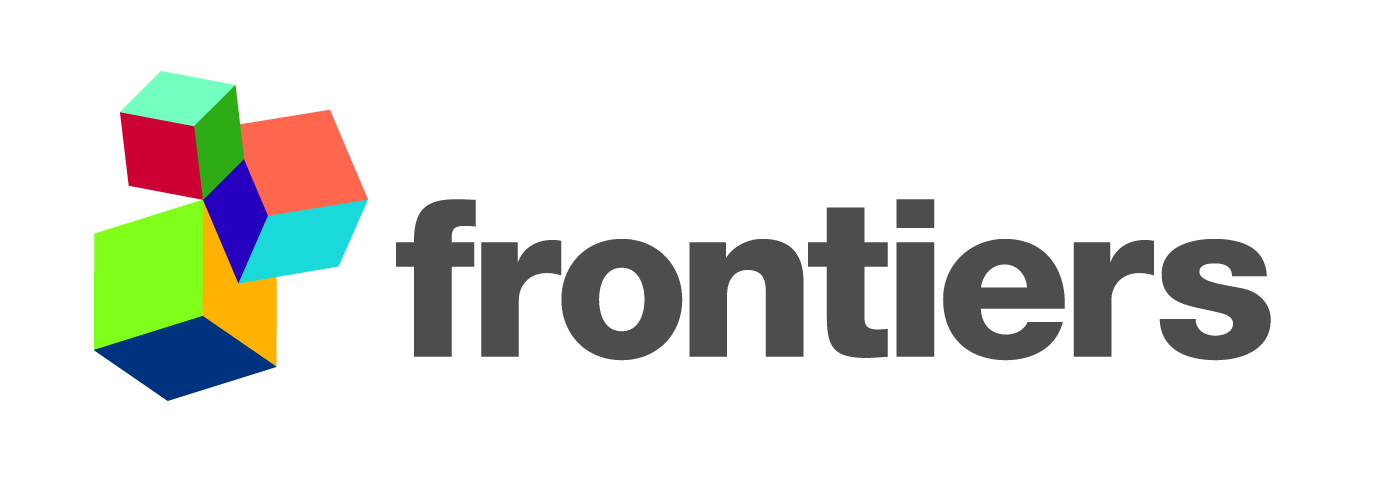


Supplementary Material

Bioluminescent bacterial assays in the assessment of seasonal and spatial patterns in toxicity of contaminated river sediments

Jarque S., Masner P., Prokeš R., Klánová J., Bláha L. *

*** Correspondence:** Corresponding Author: blaha@recetox.muni.cz

**Supplementary Table S1.** A sample of criteria for dividing parameters into 4 groups – levels of contamination. The organic carbon content (TOC) represents basic sediment characteristics, sum of polycyclic aromatic hydrocarbons (PAHs) represent non-chlorinated organic contaminants and cadmium (Cd) is a typical example of toxic metals.

|  |  |  | Group I | Group II | Group III | Group IV |  |
| --- | --- | --- | --- | --- | --- | --- | --- |
| Parameter | N^a^ | MIN0 | 〈MIN; L1〉 | (L1; L2〉 | (L2; L3〉 | (L3; MAX〉 | MAX0 |
|  |  |  |  |  |  |  |  |
| **Bottom sediment** |  |  |  |  |  |  |  |
|  |  |  |  |  |  |  |  |
| TOC (%) | 73 | 0.2 | 〈0.6; 2.2〉^b^ | (2.2; 3.8〉 | (3.8; 5.5〉 | (5.5; 7.1〉 | 7.7 |
| PAHs (μg/g) | 73 | 0.5 | 〈0.6; 3.9〉 | (3.9; 7.2〉 | (7.2; 10.5〉 | (10.5; 13.8〉 | 14.3 |
| Cd (ng/g) | 73 | 0.1 | 〈0.1; 1.2〉 | (1.2; 2.2〉 | (2.2; 3.3〉 | (3.3; 4.4〉 | 4.4 |
|  |  |  |  |  |  |  |  |
| **Recent sediment** |  |  |  |  |  |  |  |
|  |  |  |  |  |  |  |  |
| TOC (%) | 50 | 1.3 | 〈1.4; 3.3〉 | (3.3; 5.2〉 | (5.2; 7.1〉 | (7.1; 9.0〉 | 9.7 |
| PAHs (μg/g) | 51 | 1.5 | 〈1.6; 5.0〉 | (5.0; 8.4〉 | (8.4; 11.8〉 | (11.8; 15.2〉 | 80.3^c^ |
| Cd (ng/g) | 51 | 0.2 | 〈0.2; 0.4〉 | (0.4; 0.6〉 | (0.6; 0.9〉 | (0.9; 1.1 〉 | 1.1 |
|  |  |  |  |  |  |  |  |

^a^ N – number of detected values; MIN0 – the minimum from all detected values; MIN – the 2nd minimal value from all detected values; L1, L2 and L3 – the upper values for the 1st, 2nd and 3rd group; MAX – the 2nd maximal value from all detected values; MAX0 – the maximum from all detected values.

^b^ The grouping intervals can be not equal seemingly but that only is a consequence of an additional rounding of numbers.

^c^ An extreme value. That is why the 2nd minimal/maximal values were used for clustering.
