# Supplementary material for: Bioluminescent Vibrio fischeri Assays in the Assessment of Seasonal and Spatial Patterns in Toxicity of Contaminated River Sediments
Source: Front Microbiol. 2016 Nov 7;7:1738. doi: 10.3389/fmicb.2016.01738 (PMC5097916; doi:10.3389/fmicb.2016.01738)
Supplement: Supplementary file 2 [file Table2.DOCX]

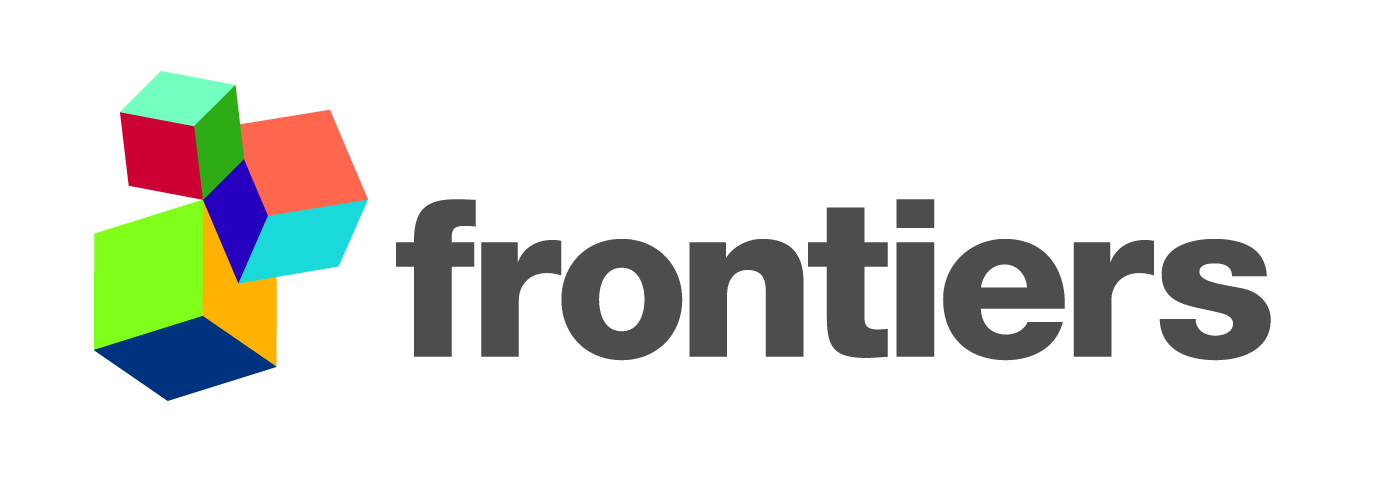


Supplementary Material

Bioluminescent bacterial assays in the assessment of seasonal and spatial patterns in toxicity of contaminated river sediments

Jarque S., Masner P., Prokeš R., Klánová J., Bláha L. *

*** Correspondence:** Corresponding Author: blaha@recetox.muni.cz

| **Supplementary Table S2:** Flash Vibrio Toxicity IC50-values in mg dw sediment/mL (95% confidence interval in parenthesses) of SUSPENSIONS of bottom sediments and fresh trapped sediments. | | | | | | | | | | |
| --- | --- | --- | --- | --- | --- | --- | --- | --- | --- | --- |
|  | toxicity (IC50, mg/ml) | | | | | | | | | |
|  | Bottom sediment - suspension | | | | | Freshly trapped sediment - suspension | | | | |
| Sampling date | Dřevnice-Malenovice | Morava-Bělov | Morava-Spytihněv | Morava-Čerťák | Čerťák-slepé rameno | Dřevnice-Malenovice | Morava-Bělov | Morava-Spytihněv | Morava-Čerťák | Čerťák-slepé rameno |
| 20/06/2007 | >75 | 63.25 (51.09-78.29) | 62.05 (54.82-70.23) | >75 | >75 | x | x | x | x | x |
| 18/07/2007 | 54.68 (42.91-69.66) | >75 | 52.69 (49.15-56.48) | >75 | >75 | 10.35 (7.84-13.67) | 42.34 (36.60-48.97) | 44.60 (38.77-51.30) | x | 48.79 (44.59-53.39) |
| 15/08/2007 | 30.63 (18.35-51.11) | >75 | 60.54 (58.17-63.01) | >75 | >75 | 27.51 (26.03-29.07) | x | 33.45 (30.40-36.80) | x | x |
| 12/09/2007 | 65.01 (55.42-76.27) | 41.92 (38.65-45.47) | 61.19 (56.46-66.31) | >75 | >75 | 37.69 (35.17-40.38) | 55.15 (46.93-64.82) | 75.76 (66.71-86.04) | x | x |
| 10/10/2007 | 36.47 (32.84-40.52) | 37.64 (35.71-39.68) | 63.17 (53.14-75.09) | >75 | >75 | 22.29 (19.14-25.95) | x | 36.30 (31.39-41.97) | x | x |
| 07/11/2007 | 60.58 (53.15-69.05) | 54.31 (47.82-61.69) | 62.12 (53.87-71.64) | 70.27 (59.42-83.37) | >75 | 20.33 (16.19-25.52) | 23.11 (19.90-26.85) | 30.51 (28.18-33.03) | 28.77 (23.70-34.94) | 18.00 (15.01-21.60) |
| 05/12/2007 | 43.80 (39.05-49.12) | 58.32 (51.59-65.93) | 46.40 (39.17-54.95) | >75 | >75 | 9.42 (7.97-11.15) | 27.55 (22.02-34.46) | 20.52 (18.30-23.01) | 21.66 (19.08-24.59) | 14.86 (13.25-16.67) |
| 02/01/2008 | 31.09 (28.14-34.45) | 50.28 (43.77-57.77) | x | >75 | >75 | x | x | x | x | x |
| 30/01/2008 | 13.53 (12.20-15.02) | 19.21 (15.50-23.81) | 73.88 (63.94-83.35) | >75 | 55.75 (48.02-64.72) | 8.11 (7.39-8.90) | 27.46 (22.99-32.80) | x | >75 | 22.91 (19.59-26.80) |
| 27/02/2008 | 16.01 (14.21-18.03) | 22.48 (17.86-28.30) | >75 | >75 | 48.01 (37.05-62.22) | 4.87 (3.49-6.79) | 21.17 (17.83-25.14) | x | 45.65 (37.36-55.77) | 14.20 (12.15-16.59) |
| 26/03/2008 | 12.35 (10.93-13.95) | 20.05 (18.25-22.03) | 38.54 (33.62-44.18) | >75 | 25.40 (18.43-35.02) | 11.03 (9.65-12.59) | x | 27.60 (24.70-30.83) | x | 19.37 (16.32-22.99) |
| 23/04/2008 | 39.40 (32.29-48.08) | 27.85 (25.43-30.50) | 53.61 (51.01-56.33) | >75 | 54.44 (50.08-59.17) | 4.62 (3.88-5.51) | x | 24.49 (21.41-28.01) | x | 17.27 (16.22-18.40) |
| 21/05/2008 | 74.06 (60.41-90.78) | 34.91 (31.70-38.45) | 58.33 (56.58-60.13) | >75 | >75 | x | 30.80 (26.45-35.86) | 29.82 (27.31-32.57) | 53.65 (45.52-63.24) | 28.40 (25.68-31.40) |
| 18/06/2008 | 73.07 (62.85-84.97) | 65.05 (61.46-68.85) | 49.08 (43.83-54.96) | >75 | >75 | 56.03 (49.70-63.17) | 42.46 (38.19-47.12) | 55.41 (49.84-61.60) | 63.65 (54.08-74.93) | 33.10 (28.48-38.46) |
| 16/07/2008 | 70.98 (65.78-76.58) | x | 63.76 (58.16-69.90) | >75 | >75 | 48.88 (41.66-57.36) | 41.93 (38.66-45.85) | 62.96 (53.48-74.13) | 45.42 (39.81-51.81) | 16.96 (14.79-19.37) |
