# Supplementary material for: Bioluminescent Vibrio fischeri Assays in the Assessment of Seasonal and Spatial Patterns in Toxicity of Contaminated River Sediments
Source: Front Microbiol. 2016 Nov 7;7:1738. doi: 10.3389/fmicb.2016.01738 (PMC5097916; doi:10.3389/fmicb.2016.01738)
Supplement: Supplementary file 3 [file Table3.DOCX]

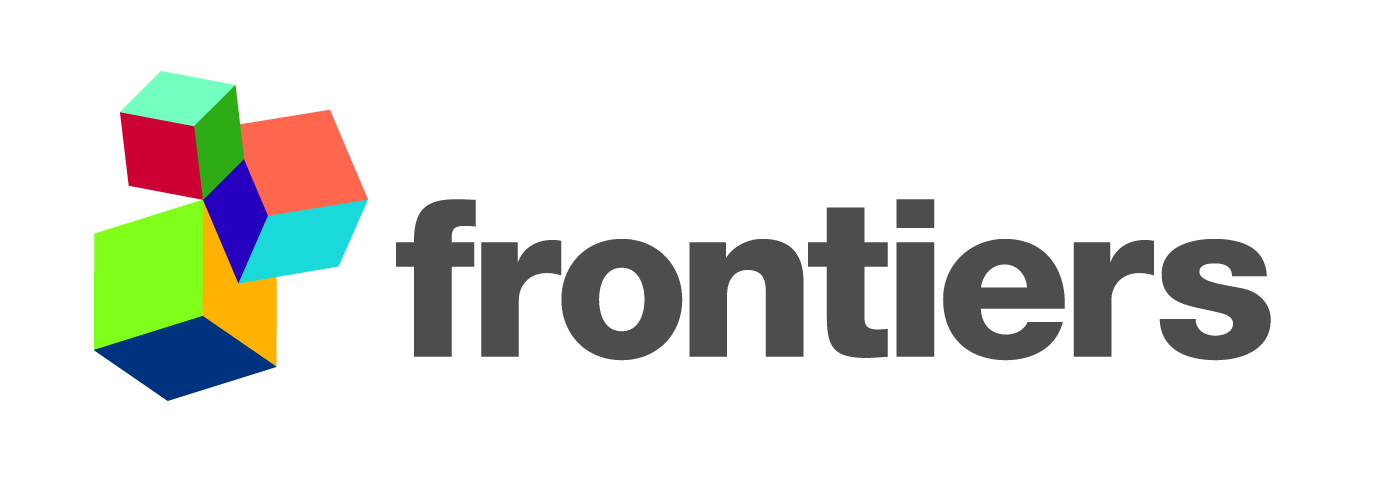


Supplementary Material

Bioluminescent bacterial assays in the assessment of seasonal and spatial patterns in toxicity of contaminated river sediments

Jarque S., Masner P., Prokeš R., Klánová J., Bláha L. *
* Correspondence: Corresponding Author: blaha@recetox.muni.cz

| **Supplementary Table S3:** Flash Vibrio Toxicity of ELUATRIATES of bottom sediments and fresh trapped sediments. Values (INH%) indicate a decrease in the light emission (% compared to control) observable after 30-s exposure in a non-diluted aqueous extract (corresponding to 75 mg dry weight sediment/ml). Higher values (i.e. higher inhibitions) indicate more pronounced toxic effects. | | | | | | | | | | |
| --- | --- | --- | --- | --- | --- | --- | --- | --- | --- | --- |
|  | toxicity (INH% at 75mg dw/mL) | | | | | | | | | |
|  | Bottom sediment - elutriate | | | | | Freshly trapped sediment - elutriate | | | | |
| Sampling date | Dřevnice-Malenovice | Morava-Bělov | Morava-Spytihněv | Morava-Čerťák | Čerťák-slepé rameno | Dřevnice-Malenovice | Morava-Bělov | Morava-Spytihněv | Morava-Čerťák | Čerťák-slepé rameno |
| 20/06/2007 | 6.45 | 9.05 | 3.60 | -3.85 | 2.30 | x | x | x | x | x |
| 18/07/2007 | 7.85 | 5.95 | 3.00 | -0.25 | 2.50 | 14.25 | 4.90 | 3.90 | x | 7.05 |
| 15/08/2007 | 6.65 | 1.75 | 2.30 | -2.40 | 3.10 | 16.40 | x | 5.30 | x | x |
| 12/09/2007 | 8.20 | 22.30 | 5.60 | 5.60 | 12.58 | 18.00 | 6.80 | 0.65 | x | x |
| 10/10/2007 | 17.55 | 23.65 | 5.05 | 3.65 | 6.30 | 35.10 | x | 7.90 | x | x |
| 07/11/2007 | 14.00 | 20.07 | 3.40 | 5.90 | 10.30 | 33.60 | 13.65 | 10.15 | 24.10 | 37.75 |
| 05/12/2007 | 19.45 | 9.20 | 12.75 | 5.25 | 6.90 | 55.25 | 13.80 | 16.05 | 44.40 | 45.15 |
| 02/01/2008 | 31.80 | 9.50 | x | 1.05 | 9.20 | x | x | x | x | x |
| 30/01/2008 | 46.15 | 29.50 | 1.15 | 0.75 | 9.05 | 49.65 | 15.65 | x | 2.80 | 28.95 |
| 27/02/2008 | 42.10 | 31.85 | 1.25 | -0.05 | 5.80 | 52.40 | 18.95 | x | 28.10 | 42.70 |
| 26/03/2008 | 51.20 | 27.90 | 14.65 | 5.60 | 9.55 | 51.60 | x | 15.20 | x | 35.75 |
| 23/04/2008 | 29.95 | 22.75 | 2.45 | 2.80 | 6.95 | 52.90 | x | 25.45 | x | 46.25 |
| 21/05/2008 | 2.55 | 15.50 | 2.80 | 1.20 | 5.15 | x | 13.55 | 11.45 | 11.00 | 18.85 |
| 18/06/2008 | 3.10 | 9.15 | 5.90 | 0.70 | 2.00 | 11.15 | 12.80 | 11.85 | 7.50 | 11.65 |
| 16/07/2008 | 0.15 | x | 5.95 | 1.75 | 1.80 | 8.35 | 5.65 | 5.35 | 11.00 | 37.50 |
